# Functional MRI of the Reserpine-Induced Putative Rat Model of Fibromyalgia Reveals Discriminatory Patterns of Functional Augmentation to Acute Nociceptive Stimuli

**DOI:** 10.1038/srep38325

**Published:** 2017-01-12

**Authors:** Jack A. Wells, Sayaka Shibata, Akihiko Fujikawa, Masayasu Takahashi, Tsuneo Saga, Ichio Aoki

**Affiliations:** 1 Department of Molecular Imaging and Theranostics, National Institute of Radiological Sciences, QST, Chiba, Japan; 2 UCL Centre for Advanced Biomedical Imaging, University College London, UK; 3 Drug Discovery Research, Astellas Pharma Inc., Japan

## Abstract

Functional neuroimaging, applied to pre-clinical models of chronic pain, offers unique advantages in the drive to discover new treatments for this prevalent and oppressive condition. The high spatial and temporal resolution of fMRI affords detailed mapping of regional pharmacodynamics that underlie mechanisms of pain suppression by new analgesics. Despite evidence supporting the translational relevance of this approach, relatively few studies have investigated fMRI abnormalities in rodent models of chronic pain. In this study, we used fMRI to map the BOLD response in a recently developed putative rat model of fibromyalgia to innocuous and acute nociceptive stimuli by applying a step-wise graded electrical forepaw stimulation paradigm, with comparison to healthy controls. We observed discriminatory functional signatures (p < 0.001) to 2 mA electrical forepaw stimulation, found to be innocuous in the control group. As such, this translational approach provides sensitive and quantitative neural correlates of the underlying chronic disease. The regional patterns of functional augmentation were found to be concordant with previous studies of nociception in the anaesthetised rat brain, supporting the specificity of this approach in the study of altered central pain processing in reserpine induced myalgia. The methodology introduced in this work represents a novel platform for emerging treatment evaluation in highly experimentally controlled conditions.

Fibromyalgia (FM) is a prevalent disorder with as yet undefined aetiology^1^. Though it lacks precise classification, FM is characterised by chronic pain and hypersensitisation together with functional symptoms such as depression, sleep disturbances and diminished mental clarity^1^,^2^,^3^,^4^. Yet, there remains a paucity of effective treatments, with many patients offered little choice but to live with the physical and emotional burden of their condition^5^. Clinical assessment of FM is hampered by reliance on self-report, confounded by the subjective perception of the individual. Therefore, there is a need for accurate, non-invasive and safe methods of pathological assessment. Functional neuroimaging studies have sought to map the neural correlates of FM in resting and acute-pain-evoked conditions (for excellent reviews see^6^,^7^,^8^). Mounting evidence identifies abnormal functional signatures associated with the disease^6^,^7^,^8^. Consequently, functional neuroimaging data can provide an objective and quantifiable assessment of the disorder.

Animal models of chronic pain that faithfully recapture properties of the human condition are highly useful for mechanistic study and therapeutic evaluation^9^. Such models facilitate longitudinal measures in experimentally controlled conditions with end-point histological examination^10^. Several previous studies have employed functional neuroimaging to map the cerebral response to acute nociceptive stimuli in anaesthetised rodents, where activation of the somatosensory cortex, cingulate cortex and thalamus are commonly observed (regions known to process afferent nociceptive signals in humans)^11^,^12^,^13^,^14^,^15^,^16^,^17^,^18^,^19^,^20^,^21^,^22^,^23^,^24^,^25^. Moreover, a recent study demonstrated correlations between the functional response to noxious electrical stimulation, measured by fMRI in the anaesthetised rat, with behavioural measures in the conscious state^26^. Collectively, these data support the translational relevance of this approach, yet relatively few neuroimaging studies have investigated functional abnormalities in animal models of chronic pain^12^,^18^,^27^,^28^,^29^,^30^. Therefore the question remains: can fMRI provide a sensitive correlate to neuropathology in rodent models of chronic pain?

We have developed a non-surgical putative rat model of FM which reflects key symptoms of the human condition, including tactile allodynia and depression^31^. The model is generated by repeated injection of reserpine, causing biogenic amine depletion (observed in CNS samples from FM patients). In this study we use fMRI to characterise the regional blood oxygen level dependant (BOLD) response to acute innocuous and noxious electrical forepaw stimuli in the reserpine induced myalgia (RIM) model, with comparison to healthy control animals. We report regionally specific and sensitive measures of functional augmentation in brain regions implicated in nociceptive processing. As such, this study provides a novel and translational methodological platform to study the neurological underpinnings of RIM-induced tactile allodynia and provides a new objective index for drug development.

## Results

### Animal Physiology

Blood gasses were sampled directly before and after fMRI acquisitions and were maintained within physiological range: Control mean (SD): pCO_2_ = 38 (±2) mmHg; pO_2_ = 109 (±5) mmHg; RIM mean (SD): pCO_2_ = 33 (±3) mmHg; pO_2_ = 118 (±9) mmHg. Blood pressure was measured throughout functional imaging: Control mean (SD) = 108/73 (±8/±5) mmHg; RIM mean (SD) = 97/69 (±10/±6) mmHg. In addition the average blood pressure change during “mild”, “moderate” and “high” electrical forepaw stimulation was recorded: Control mean (SD): “mild” = 0 mmHg (±0), “moderate” = 1 (±1), “high” = 7 (±4) mmHg; RIM mean (SD): “mild” = 0 mmHg (±0), “moderate” = 0 mmHg (±0), “high” = 3 (±1) mmHg. The von Frey tests in the RIM subjects returned a mean (SD) of 1.73 (±0.83) g, concordant with our previous characterisation of the RIM model^31^. At the time of imaging, the mean weight of the control and RIM animals was 267 (±8) g and 192 (±11) g respectively. The mean BOLD fMRI signal within the SSFP region at baseline was 132(±7) and 137(±14) [arbitrary units] and the temporal SNR at baseline (mean/sd) was 504(±106) and 518(±158) for the normal and RIM cohorts respectively.

### fMRI of “Moderate” Electrical Forepaw Stimulation is a Sensitive Marker of Reserpine Induced Myalgia

**Figure 1:**
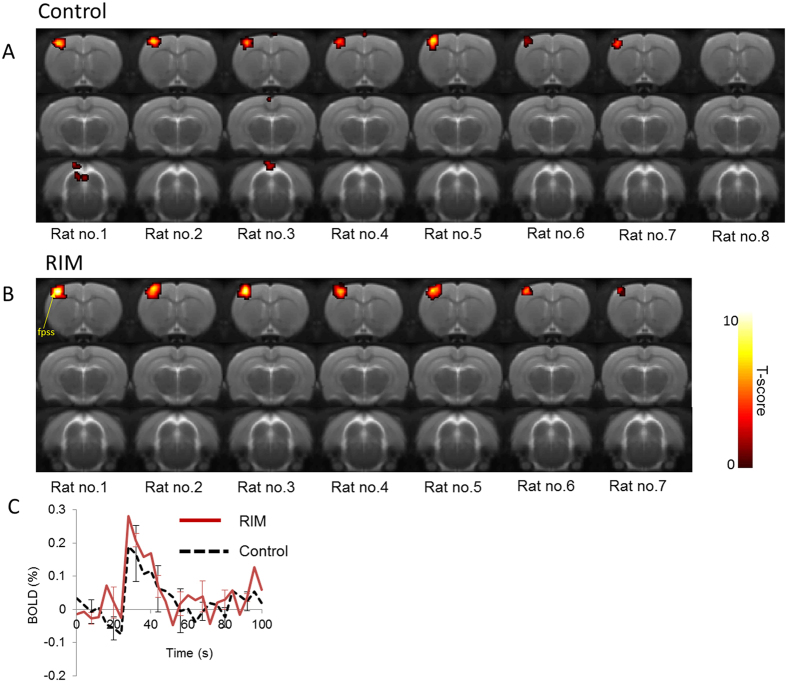
BOLD activation maps to “mild” electrical forepaw stimulation across 3 coronal slices within each of the individual subjects for the control (**A**) and RIM (**B**) cohort (p < 0.001, nv = 5). The activation maps are overlaid on a T2-weighted MRI rat brain atlas. The BOLD timeseries data within the anatomically defined forepaw region of the somatosensory cortex (fpss) is also shown (**C**).

Figure 1(A,B) shows BOLD activation maps to “mild” electrical forepaw stimulation in three coronal slices from each of the individual animals in the control and RIM cohorts. Mild forepaw stimulation (1 mA) resulted in consistent BOLD signals that were isolated to the unilateral forepaw region of the somatosensory cortex (fpss) in all control and RIM subjects. These data indicate that 1 mA stimulation is innocuous in both groups. The BOLD signal timecourse data within the fpss was highly similar between control and RIM subjects (p = 0.78, Fig. 1C).

**Figure 2:**
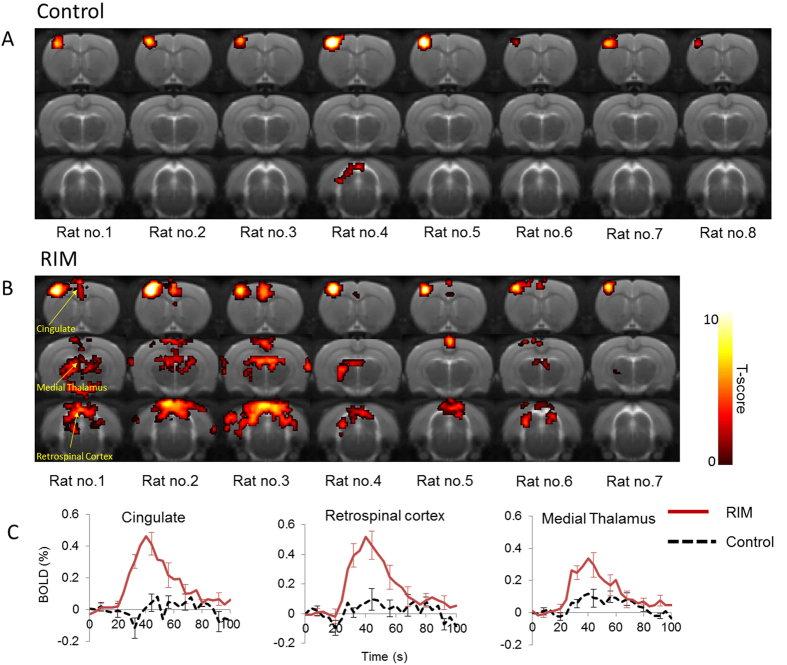
BOLD activation maps to “moderate” electrical forepaw stimulation across 3 coronal slices within each of the individual subjects for the control (**A**) and RIM (**B**) cohort (p < 0.001, nv = 5). The activation maps are overlaid on a T2-weighted MRI rat brain atlas. The BOLD timeseries data within the anatomically defined regions are also shown (**C**).

In the same animals, “moderate” stimulation (2 mA) evoked widespread, regionally specific BOLD signals in the RIM cohort with activation still confined to the fpss in the control group (Fig. 2). Visual inspection of the individual animal BOLD SPMs indicated activation of the cingulate cortex and retrosplenial cortex (in 6 of 7 RIM subjects), and medial/dorsal thalamus (in 5 of 7 RIM subjects). Interestingly, a relatively small region of BOLD activation is present in the cingulate of RIM subjects 4 and 5 within the deep cortical layers. This observation may reflect the relative density of nociceptive neurons in the rat cortex which is greatest in layer 5^16^,^32^. Within these regions, BOLD signal changes above baseline were observed in the RIM group, with the fMRI signal during stimulation often indistinguishable from baseline measurements in the control rats (Fig. 2C). The mean BOLD signal change in the cingulate cortex, retrosplenial cortex and medial dorsal thalamus was significantly greater in the RIM group in comparison to the controls (p = 0.0003, 0.0003 and 0.02 respectively). However no significant changes in the BOLD response were observed in the fpss (Fig. 2C). This finding mirrors clinical observations where matched nociceptive stimuli in FM patients illicits greater functional outcomes in regions implicated in pain processing, relative to healthy controls^6^. The data presented in Fig. 2 highlights the sensitivity of fMRI to detect tactile allodynia, induced by the underlying chronic pathology, in the RIM model (previously characterised by behavioural measures^31^). Thus, even with a comparatively small cohort, highly significant differences can be detected in regions implicated in nociceptive processing due to the reproducibility and sensitivity of the methodology at “moderate” stimulation.

**Figure 3:**
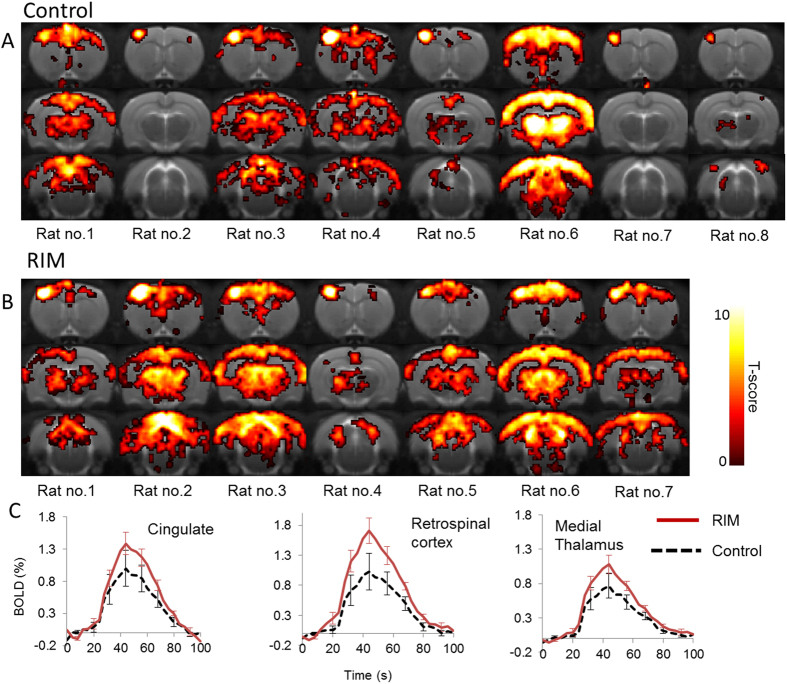
BOLD activation maps to “high” electrical forepaw stimulation across 4 coronal slices within each of the individual subjects for the control (**A**) and RIM (**B**) cohort (p < 0.001, nv = 5). The activation maps are overlaid on a T2-weighted MRI rat brain atlas. The BOLD timeseries data within the anatomically defined regions are also shown (**C**).

As the electrical stimulus was further increased to 3.8 mA, widespread bilateral activation was observed in both control and RIM groups (Fig. 3). However, visual inspection suggested greater inter-animal variability relative to “mild” and “moderate” stimulus, in both cohorts. Indeed, although the average BOLD response in the cingulate cortex, medial dorsal thalamus and retrosplenial cortex was greater in the RIM cohort, these were no longer significant.

### Spatial Patterns of Functional Augmentation to “Moderate” Stimulation are Specific to the Nociceptive Pathway

**Figure 4:**
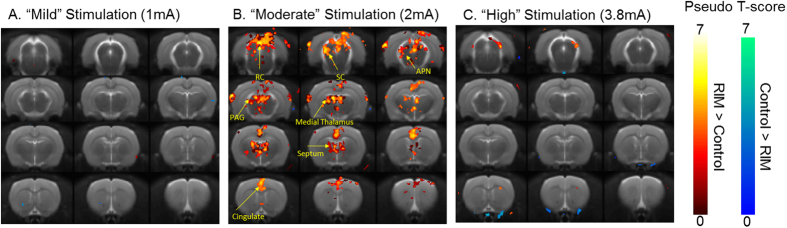
Group-wise BOLD activation maps for non-parametric random effects analysis: for “mild” (**A**), “moderate” (**B**) and “high” (**C**) electrical forepaw stimulation. The activation maps are overlaid on a T2-weighted MRI rat brain atlas. Significantly greater activation in the RIM group relative to the control group is shown by a red/yellow colourbar and significantly greater activation in the control group relative to the RIM group is shown by a blue/green colourbar. Abbreviations: RC = retrosplenial cortex; SC = superior colliculus; APN = anterior pretectal nucleus. Threshold = P < 0.001 with a cluster size of 5 voxels.

A two-group random effect design was employed to investigate voxel-wise differences in BOLD contrast changes due to forepaw stimulation between control and RIM animals at each of the applied electrical forepaw stimulus intensities. Figure 4 shows the resultant statistical non-parametric maps. In concordance with the individual animal data presented in Fig. 2, “moderate” stimulation resulted in increased BOLD responses in the RIM cohort within brain regions implicated in previous studies of acute nociception in the anaesthetised rat brain (including cingulate cortex, retrosplenial cortex, medial dorsal thalamus). In contrast, negligible group differences were observed at “mild” and “high” stimulation in agreement with the individual animal data presented in Figs 1 and 3.

### Histology

Supplementary Figures 1–3 shows histological sections of the normal and RIM rat stained with H&E, GFAP, Caspase-3 and IBA1. Visual assessment of these data indicates that the augmented fMRI response to forepaw stimulus is not confounded by gross structural (H&E) and/or cellular (GFAP: astrogliosis, Caspase-3: apoptosis, and IBA1: microgliosis) differences between the control and RIM model.

## Discussion

After more than 10 years since the first fMRI study in FM patients^33^, this is the first “back-translational” investigation of a putative animal model of the condition using functional neuroimaging techniques. Common symptoms of FM include persistent pain, cognitive impairment and depression. However, it is the fMRI signature to acute nociceptive stimuli that has shown most promise as a specific and quantitative clinical biomarker of FM, distinguishing it from other chronic pain disorders^7^,^34^. Here, we investigated whether this functional correlate presented in a recently developed putative rat model of the condition^31^. “Moderate” electrical stimulation evoked widespread and regionally discrete BOLD activations, a sensitive measure of the underlying neuropathology (no such activation patterns were observed in the control cohort (Fig. 2)). The differential patterns (Fig. 4) of fMRI signals between the RIM and control animals were in close agreement with previous studies of nociceptive electrical stimulus in the anaesthetised rat brain^11^,^16^. This suggests that the functional augmentation observed in the RIM model at “moderate” stimulation is specific to nociceptive processing. Moreover, histological analysis identified no marked structural or cellular difference. These findings lend support to the translational relevance of the RIM model^35^ within a novel methodology that may be valuable in the quantitative assessment of emerging therapeutic options for FM.

Despite prior clinical investigations, the mechanisms that trigger and sustain FM are poorly understood^7^. Hence it remains a condition defined by symptoms and not underlying pathology^36^. The lack of peripheral pathology presents a significant hurdle to the generation of an animal model of the disease, with no discrete target for pathological induction (unlike spinal cord injury, for example^18^). Based on recent evidence for the role of dopamine in pain and analgesia within the CNS^37^, Nagakura and colleagues generated the RIM model by disrupting the biogenic amine system with repeated injection of reserpine^31^. Biogenic amine depletion in the CNS induced tactile allodynia and depressive like behaviour^31^. Importantly, similar responses in the RIM model were observed to treatments tested in FM patients. Though it is yet to be demonstrated that the RIM model comprehensively reflects the multifaceted symptoms in FM patients^35^, it represents a progressive step in the study of the underlying causes of clinical symptoms and their response to treatment^36^.

To our knowledge, this is the first report of functional activation of the nociceptive pathway during 2 mA electrical stimulus, normally considered to be innocuous in anaesthetised rats^16^ (indeed consistent activation was confined to the forepaw region of the somatosensory cortex (ssfp) in healthy control subjects [Fig. 2]). This observation highlights the non-linear stimulus-response relationship evident in studies of cerebral pain processing. As such, application of “moderate” acute stimulus intensity (below the threshold of nociception in the healthy controls) may be advantageous to better discriminate the healthy and RIM condition, as found in the present study (Figs 1, 2, 3 and 4). The patterns of differential BOLD activations to “moderate” stimulus between the RIM and control groups (Fig. 4) are in close agreement with a recent fMRI study of electrical forepaw stimulation in medetomidine/isoflurane anaesthetised rats^11^. In both studies, activation of thalamic regions, S1, cingulate, retrosplenial cortex, superior colliculus and periacqueductal gray was observed but not the amygdala or insula (regions implicated in pain processing in humans). The lack of BOLD response in these regions may reflect inter-species differences, the mode of nociceptive challenge or potential confounds associated with anaesthesia, inherent limitations of this approach. In contrast to recent experiments under similar conditions^11^, we found no evidence for negative BOLD responses in the caudate putamen. This may reflect the different anaesthetic regimes, or the greater stimulus strength applied in the earlier study. Negligible differences in the BOLD response between the control and RIM groups were observed at “mild” or “high” electrical paw stimulation (Figs 1, 3 and 4). In this case of “mild” stimulation this is likely because this stimulus does not exceed the pain threshold for either group under these experimental conditions. This also provides evidence that animal physiological and neurovascular coupling was highly similar between the groups. In the case of “high” stimulation, visual inspection of Fig. 3 suggests that this there is a trend for increased BOLD response in the RIM cohort relative to the control cohort. However the inter-subject variability of the responses in both cohorts is markedly greater relative to “moderate” stimulation (Fig. 2). Therefore, from a methodological perspective we recommend future studies consider using “moderate” rather than “high” stimulation due to the improved reproducibility of the data at “moderate” stimulation relative to “high” stimulation.

fMRI studies of FM patients have found significant increases in the BOLD response to the same acute painful stimuli (relative to healthy volunteers), in the SI/SII, anterior cingulate cortex, insula, posterior cingulate cortex, superior temporal gyrus, cerebellum and inferior parietal lobule^33^. A later study measured an increased functional response to heat stimulus in the prefrontal, supplemental motor, insular, and anterior cingulate cortices^38^, findings later confirmed in a different cohort of patients^39^. A more recent study reported augmented fMRI signals in the front-cingulate cortex, supplementary motor areas and thalamus^40^. Similarly we observe increased BOLD response in the cingulate and thalamic regions, in response to “moderate” electrical stimulation in the RIM cohort relative to healthy controls. Interestingly, a later fMRI study, using matched perceived painful stimulus, observed reduced BOLD responses in the thalamus rostral ACC and brainstem in FM patients compared to controls which they interpreted as evidence for impaired inhibition of pain processing in patients^41^. However we did not implement this experimental design because of the difficulty in applying matched perceived electrical stimulus to the anaesthetised rodent. An earlier study found evidence for altered descending pain control mechanisms in FM sufferers by measuring differential BOLD responses in the spine and brainstem relative to normal controls^42^. Our fMRI sequence did not capture these regions which may merit future investigation given the findings by Bosma *et al*.,^42^. There is evidence that FM patients also exhibit altered temporal BOLD response profiles^43^ and examination of this property in the RIM model would represent an interesting extension of this work, though beyond the scope of the current investigation. A recent study used fMRI to link the endogenous opioid system to regional pain-evoked brain activity in Fibromyalgia patients^44^. The methods presented in the current study may provide a platform for further investigation of functional and molecular interactions, given the use of invasive measures in animal models, not normally possibly in humans.

The fMRI measurements in this study were performed in the anaesthetized brain (1.0% isoflurane), which may confound the physiological relevance of data interpretation. Indeed a fMRI study in the awake rat revealed more widespread patterns of BOLD signal changes in response to noxious heat stimuli (though contributions of motion and stress cannot be easily dissected)^45^. Importantly, a recent study observed correlations between fMRI measures to noxious stimuli in the medatomonine/isoflurane anaesthetized rat with vocal responses to nociceptive stimuli in the awake animal^26^. These data suggest that fMRI in the anaesthetized brain does provide a physiologically relevant correlate of acute nociception. Therefore, although anaesthesia undoubtedly influences fMRI signals, provided there is a degree of BOLD activation driven by nociceptive processing, useful information can still be extracted regarding the effect of new analgesics in the CNS. It should be noted that the mean pCO_2_ is slightly higher in the control group relative to the RIM group (38 vs 33 mmHg). However, the pCO_2_ in both groups is still within physiological range and is unlikely to account for the observed differences in BOLD response between the groups. Indeed we observed no correlation between the pCO_2_ and BOLD response across the 15 animals studied in this work within the ssfp regions at any of the stimulation strengths (data not shown). Furthermore the BOLD response to innocuous “mild” stimulation were highly similar between the RIM and control groups suggesting that possible respiratory differences do not account for the between-group differences observed at “moderate” stimulation.

To conclude, in this study we investigated the functional response to acute innocuous/noxious stimuli in the reserpine induced rat model of FM using fMRI. We observed discriminatory patterns of BOLD activations to “moderate” stimulation (found to be innocuous in the control group). This finding reveals the regional functional augmentation that underlies behavioural measures of tactile alloydinia in the RIM model, and mirrors observations in FM patients. Given the sensitivity and reproducibility of the fMRI measures applied to the non-surgical model, we suggest that this method may be highly advantageous for drug development prior to clinical assessment.

## Methods

### Reserpine Induced Myalgia Model

All animal work included carefully applied endpoint definition (see below) and was approved by the Institutional Animal Care and Use Committee of the National Institute of Radiological Sciences (Chiba, Japan – no. 13–1008). All the experiments in this study were carried out in accordance with the Institutional Animal Care and Use Committee of the National Institute of Radiological Sciences (Chiba, Japan – no. 13–1008). The endpoint definition was as follows: i) observation of difficulty in breathing, spasm, self-mutilation or abnormal movement; ii) more than 20% body-weight loss per week; iii) the RIM model must be euthanized within 3 weeks after the final administration of reserpine or directly after fMRI experiments (non-recovery). All animal models were carefully bred and observed by animal technologists and no animal deaths were observed during the study. The RIM model was generated as described previously^31^. Reserpine (30013–81, Nakarai, Japan) was injected subcutaneously (1 mg/ml [with 0.5% acetic acid], 1 mg/kg) per day for three days. RIM rats were imaged within 14 days of the initial reserpine injection. Control (with no reserpine injections (n = 8)) and RIM (n = 7) imaging experiments were performed in an interleaved manner. In 3 of the 7 RIM animals, von Frey filament testing (applied to the plantar surface of the hindpaw) was performed as a behavioural measure of tactile allodynia. Please refer to our previous work^31^ for a detailed description of the behavioural characterisation of the RIM model.

### Animal Preparation

Prior to imaging, anaesthesia was induced and maintained on 2.0% isoflurane (Escain, Mylan Co. Ltd., Tokyo, Japan). The femoral vein and artery were cannulated using polyethylene catheters (PE-50, Becton Dickinson, MD, USA) for drug administration, monitoring of blood pressure and blood gas sampling. The rat was intubated to allow ventilation (MRI-1, MRI compatible ventilator, CWE Inc., PA, USA) during image acquisition. The animal was then transferred to the scanner bed where 2 needle electrodes were implanted subcutaneously between the second and fourth digit of the right forepaw. Forepaw muscle twitching was visually confirmed prior to subsequent infusion of muscle relaxant. Once the animal was transferred to the centre of the MRI scanner bore, isoflurane concentration was reduced to 1.0% in air/oxygen and the muscle relaxant gallamine ([50 mg/ml], 0.3 ml/hr) was infused into the femoral vein. During imaging, animal temperature was maintained at 36.5–37.5 °C using heated water tubing and a hot air fan.

### FMRI Acquisition and Analysis

Images were acquired using a small animal 7 T MRI scanner (20-cm bore diameter, Biospec AVANCE-III system, Bruker Biospin, Germany). Signal transmission and reception was achieved using a single loop surface coil positioned dorsal to the head. Following acquisition of scout images, an automated field-mapping based shimming procedure was performed over the imaging volume (typical linewidth [FWHM = 13–15 Hz]). Functional images were acquired using a 4 shot segmented GE-EPI sequence with the following parameters: TE = 15 ms, TR = 500 ms, 2 averages [temporal resolution = 4 s (4 shot, TR = 500, 2 averages)], matrix size = 96 × 96, field of view = 38.4 × 38.4 mm, 13 slices, slice thickness = 1 mm, slice gap = 0 mm. The 13 coronal slices (acquired in an interleaved manner) were manually positioned to contain the rostral edge of the pre-frontal cortex to immediately rostral to the cerebellum. Blood gasses were sampled prior to fMRI acquisitions (i-STAT, Abbott Point of Care Inc., NJ, USA) and ventilation parameters were adjusted until normal physiological range was achieved. Blood gasses were additionally sampled at the end of fMRI data collection. Blood pressure and core temperature were monitored continuously throughout imaging (MP150, Biopac systems Inc., CA, USA). Typical ventilation parameters were 70 breaths/min and 2.8 ml tidal volume.

In order to investigate BOLD changes in response to electrical forepaw stimulation, a standard ‘task-based’ block design approach was used that consisted of 20 s forepaw stimulation (10 Hz, 0.5 ms pulse width) with 2 minutes inter-stimulus-interval, repeated 5 times for a total imaging time of ~11 minutes. The current of the electrical forepaw stimulation was modulated (1 mA, 2 mA, 3.8 mA – defined as “mild”, “moderate” and “high”) for each fMRI timeseries. Each of these fMRI time-series (consisting of the block design described above) at constant electrical forepaw stimulation current were run three times in an interleaved manner [i.e. fMRI time-series 1 (1 ma); fMRI time-series 2 (2 ma); fMRI time-series 3 (3.8 ma); fMRI time-series 4 (1 ma); fMRI time-series 5 (2 ma); fMRI time-series 6 (3.8 ma); fMRI time-series 7 (1 ma); fMRI time-series 8 (2 ma); fMRI time-series 69(3.8 ma)].

In a single control animal, artefacts were present in the GE-EPI images in one of the three fMRI experiments at 2 mA stimulation current, due to RF interference. These data were excluded from the analysis. No other acquired data in the 15 subjects was excluded and data analysis was performed using an automated pipeline in the statistical parametric mapping (SPM) software (http://www.fil.ion.ucl.ac.uk/spm/ -described immediately below).

For each subject, fMRI timeseries data at each of the three different applied electrical forepaw stimulus intensity values were averaged and registered to a rat brain atlas^46^,^47^ using SPM automated registration tool. The relatively high resolution and minimal distortion in the fMRI images facilitated accurate registration of the functional data directly to the atlas (confirmed by visual inspection). Images were then spatially smoothed (0.5 mm FWHM Gaussian kernel), and first level analysis of each time-series using an on/off regressor derived from the applied forepaw stimulus paradigm (and convolved with the standard HRF (SPM)) was applied to generate a statistical map at each forepaw stimulus current intensity for each subject. In order to visualise the individual subject BOLD activation maps to forelimb stimulation, a minimum threshold of p < 0.001 with a cluster size of >5 voxels was chosen. WFU pickatlas (http://fmri.wfubmc.edu/software/pickatlas) and the marsbar toolbox (http://marsbar.sourceforge.net/) were used to extract the BOLD timeseries data from anatomically defined ROIs that were delineated from the parcellated rat brain atlas^46^,^47^. From the raw BOLD time series data, the percentage change in the fMRI signal during forepaw stimulation (relative to the mean baseline signal 20 s before the stimulation) was calculated and then averaged across all the repeated periods of stimulation (for each stimulus strength separately). This was then converted to an average time-series plot across all subjects with the error bars representing the standard error of the mean across the subjects (shown in Figs 1, 2 and 3).

To investigate possible differences in the magnitude of the BOLD signal within the ROIs (taken from the atlas and not from the “activated” regions or by manual placement), the mean BOLD signal % change from baseline during 20 s stimulation was calculated for each subject/forepaw stimulation intensity. Possible differences in the mean BOLD % change between the RIM and control subjects were investigated using Mann-Whitney (a non-parametric test was chosen given the sample size [8 control and 7 RIM rats]).

A two group random effects voxel-wise analysis was performed to examine differences between the BOLD activation to forepaw stimulation in the control vs RIM cohort. The statistical non-parametric mapping toolbox was used (http://warwick.ac.uk/snpm) with a minimum threshold of p < 0.001 and a cluster size of >5 voxels. After the MRI acquisitions, all animals were euthanized with a pentobarbital injection via femoral vein.

### Histology

Histological analysis was performed in a single control and RIM subject to investigate possible structural changes in the RIM model. Three coronal slices were taken at bregma location +1.08, −5.05, −6.72 mm and the following immunohistochemistry staining was performed: hematoxylin and eosin (H&E); glial fibrillary acidic protein (GFAP); caspase-3; ionized calcium-binding adapter molecule (Iba1). All histological data are reported in Supplementary Figures 1–3.

## Additional Information

**How to cite this article**: Wells, J. A. *et al*. Functional MRI of the Reserpine-Induced Putative Rat Model of Fibromyalgia Reveals Discriminatory Patterns of Functional Augmentation to Acute Nociceptive Stimuli. *Sci. Rep.* **7**, 38325; doi: 10.1038/srep38325 (2017).

**Publisher's note:** Springer Nature remains neutral with regard to jurisdictional claims in published maps and institutional affiliations.

## Supplementary Material

Supplementary Dataset 1
