# Supplementary material for: Functional MRI of the Reserpine-Induced Putative Rat Model of Fibromyalgia Reveals Discriminatory Patterns of Functional Augmentation to Acute Nociceptive Stimuli
Source: Sci Rep. 2017 Jan 12;7:38325. doi: 10.1038/srep38325 (PMC5228122; doi:10.1038/srep38325)

**Supplementary Figures: Functional MRI of the Reserpine-Induced Putative Rat Model of Fibromyalgia Reveals Discriminatory Patterns of Functional Augmentation to Acute Nociceptive Stimuli**

Jack A Wells, Sayaka Shibata, Akihiko Fujikawa, Masayasu Takahashi, Tsuneo Saga, Ichio Aoki


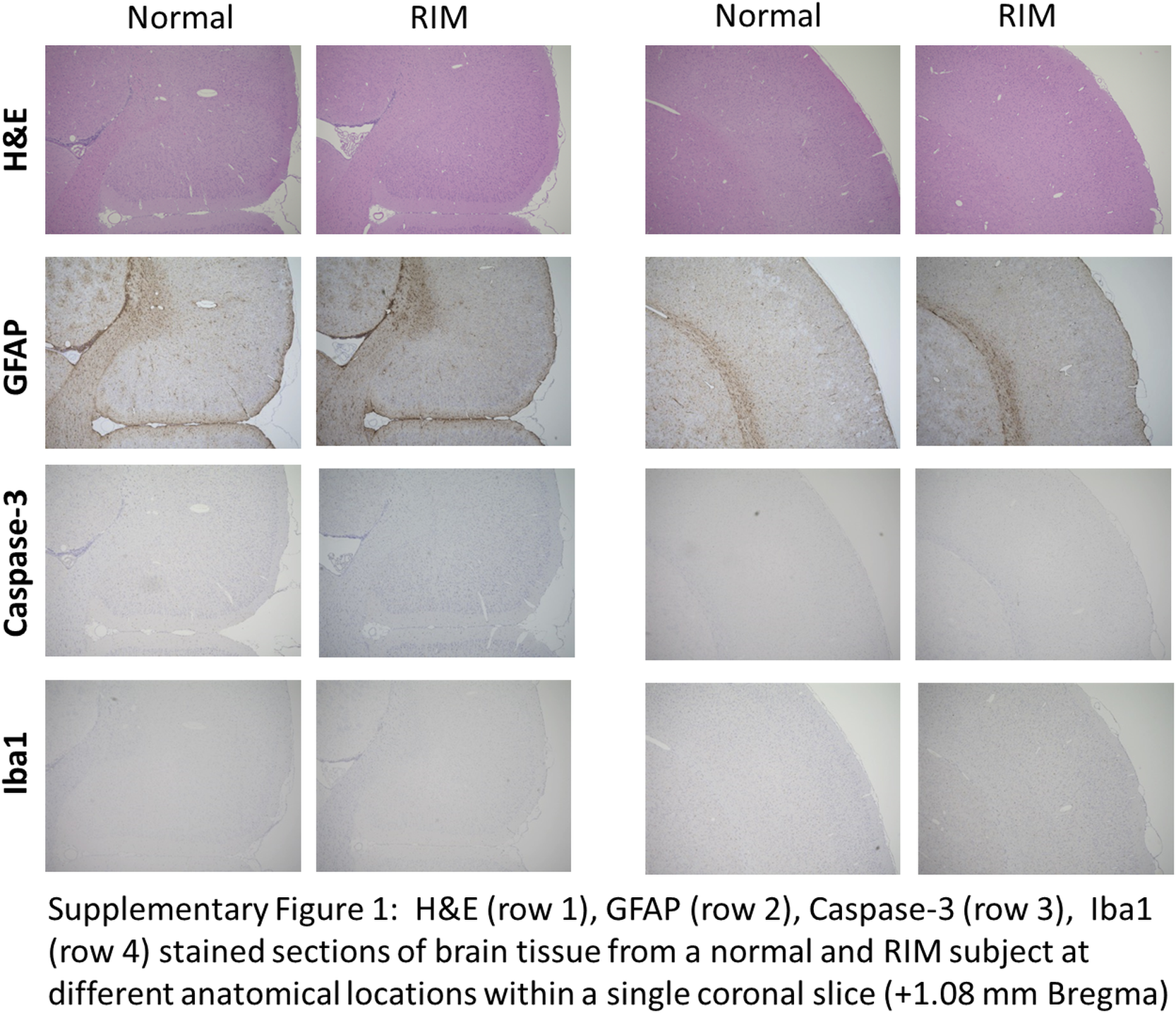


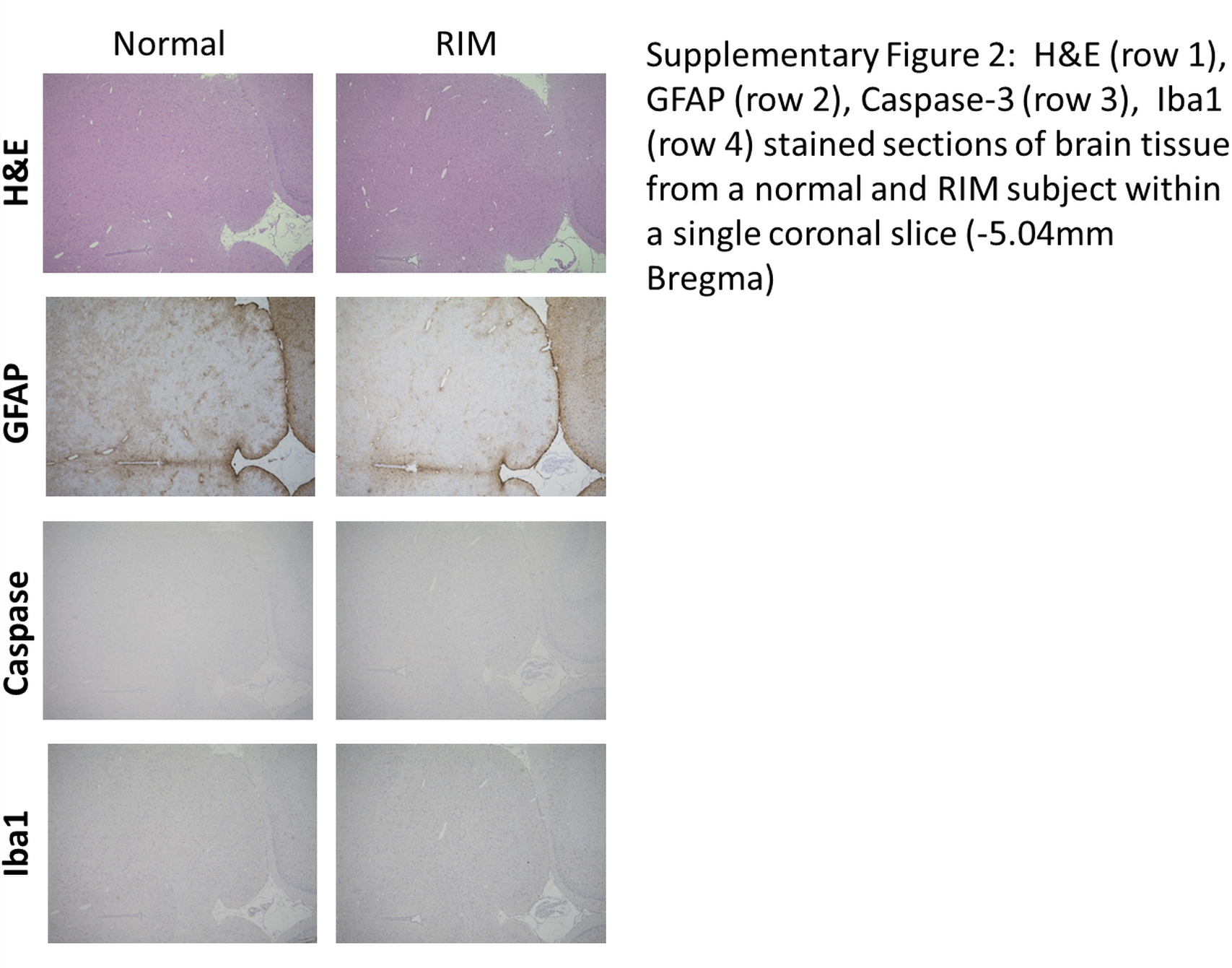


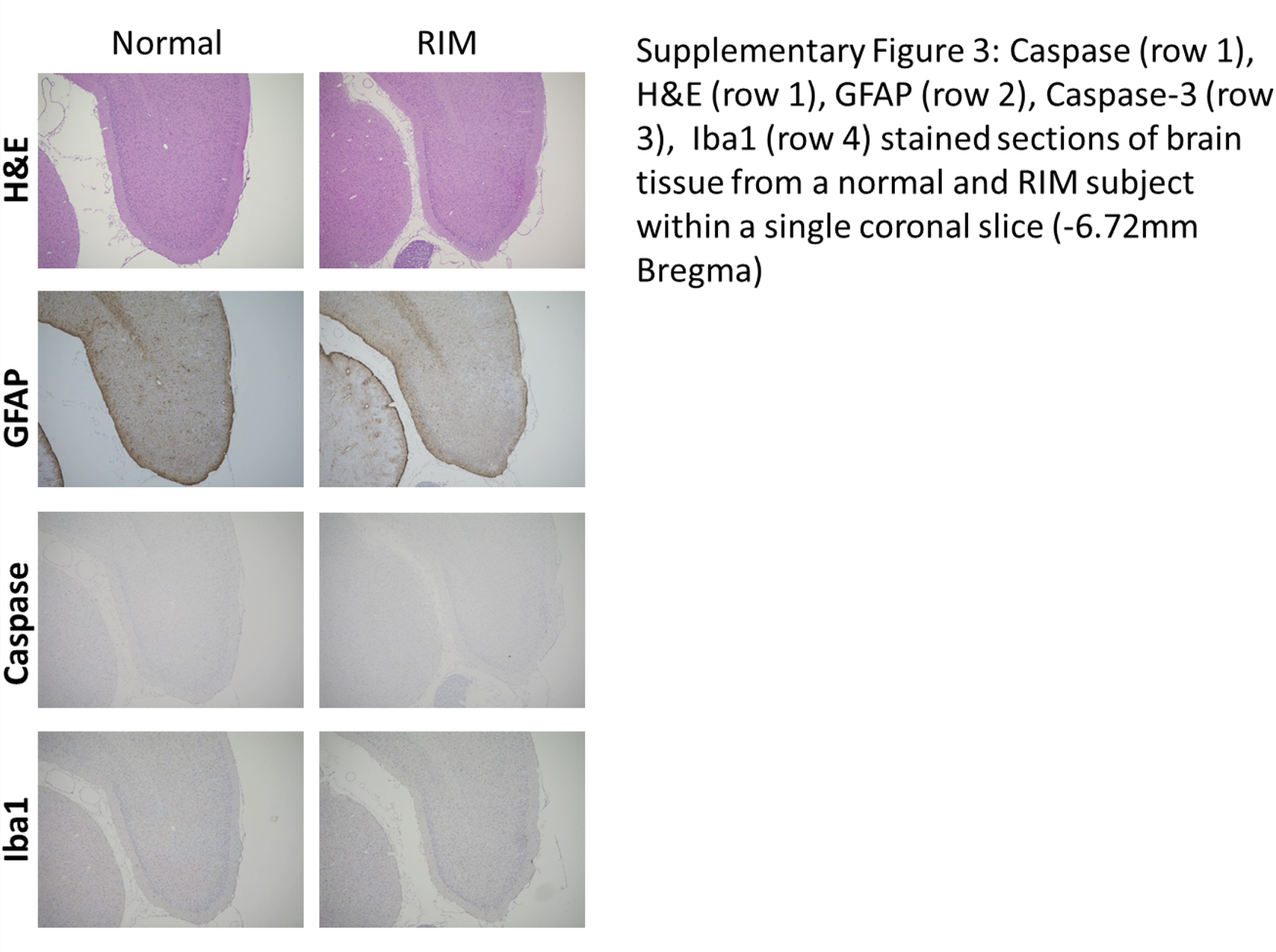

Supplement: Supplementary Dataset 1 [file srep38325-s1.doc]
